# Treatment of polypoidal choroidal vasculopathy by photodynamic therapy, aflibercept and dexamethasone triple therapy

**DOI:** 10.1038/srep36870

**Published:** 2016-11-16

**Authors:** Mary Ho, Donald C. F. Woo, Vesta C. K. Chan, Alvin L. Young, Marten E. Brelen

**Affiliations:** 1 Department of Ophthalmology and Visual Sciences, Prince of Wales hospital, Hong Kong, China; 2 Hong Kong Ophthalmic Associates, Mong Kok, Kowloon, Hong Kong, China; 3 Department of Ophthalmology and Visual Sciences, The Chinese University of Hong Kong, Hong Kong, China

## Abstract

Polypoidal choroidal vasculopathy is a relatively common type of degenerative macular disease among the Chinese population. This study aims to describe the therapeutic responses to combination therapy with photodynamic therapy, intravitreal aflibercept and intravitreal dexamethasone in patients with polypoidal choroidal vasculopathy. A prospective series of 17 eyes of 13 patients suffering from treatment-naïve polypoidal choroidal vasculoapathy were recruited. All cases received triple therapy with photodynamic therapy, intravitreal aflibercept and intravitreal dexamethasone and one year outcomes were reported. The baseline visual acuity was 0.65logMAR +/− 0.38 (Snellen 20/80 to 20/100). The visual acuity at 1 week, 3 months, 6 months and one year after treatment were significantly improved to 0.522logMAR+/− 0.365 (P < 0.04) (Snellen 20/70), 0.363logMAR+/−0.382 (Snellen 20/50;P < 0.001), 0.377logMAR +/− 0.440 (Snellen 20/50;p = 0.005), and 0.35logMAR +/− 0.407 (Snellen 20/40;P < 0.001), respectively. The baseline central foveal thickness (CFT) on optical coherence tomography (OCT) was 394.7 +/− 70.6 μm. CFT at 6 months and 1 year after treatment were significantly reduced to 259 +/− 54 μm (p = 0.004) and 271 +/− 49.7 μm(p = 0.016), respectively. Triple therapy with photodynamic therapy, intravitreal aflibercept and intravitreal dexamethasone is an effective treatment for polypoidal choroidal vasculopathy. The majority of cases responded well with significant responses observed as early as 1 week after initiation of therapy.

Polypoidal choroidal vasculopathy (PCV) is an exudative maculopathy with a high prevalence in Asia^1^. Between 10–54% of Asian patients presenting with age-related macular degeneration are reported to have PCV^2^. The gold standard for diagnosing PCV is by indocyanine green angiography (ICG) which shows the presence of a branching choroidal vascular network and/or clusters of dilated polypoidal lesions^3^,^4^.

The treatment options for PCV include anti-vascular endothelial growth factor (VEGF) and verteporfin photodynamic therapy (PDT). Anti-VEGF agents improve visual function by restoring normal retinal thickness while PDT facilitates polyp regression. The efficacy of these treatments as monotherapy versus combination therapy was previously evaluated in a randomized controlled trial (EVEREST)^5^. The results showed that PDT, used in combination with ranibizumab or used alone, is superior to ranibizumab monotherapy in achieving polyp regression. The rate of polyp regression was 77.8% with combination therapy, 71.4% with PDT alone and 28.6% with ranibizumab monotherapy. Complete regression of polyps is desirable during treatment of PCV, since the persistence of polyps significantly increases the risk of recurrent haemorrhage and re-accumulation of subretinal fluid^6^. Hence, PDT is considered an essential component in the treatment of PCV despite its potential side effects which include retinal pigment epithelial (RPE) rips, subretinal haemorrhage, scarring or atrophy which may lead to long term visual impairment^7^,^8^.

Aflibercept is an anti-angiogenic agent that blocks VEGF-A, VEGF-B and placental growth factor^9^,^10^. Given the ability of aflibercept to block multiple angiogenic factors and its high VEGF-binding affinity^9^,^11^, it is currently used for choroidal neovascularization secondary to various causes^12^. Multiple studies have shown potential benefits of switching to aflibercept in treatment-resistant cases of AMD^13^,^14^,^15^,^16^,^17^,^18^,^19^. Aflibercept has also been reported to be effective in treating polypoidal choroidal vasculopathy as monotherapy without PDT^20^,^21^,^22^,^23^. However, monotherapy requires repeated aflibercept injections, resulting in cost implications for patients who need to pay for the injections themselves.

Intraocular inflammatory cytokines are likely to be elevated in eyes with PCV especially in the presence of subretinal haemorrhage or macular edema. PDT application may further perpetuate the intraocular inflammation. Intravitreal corticosteroids may therefore be useful in the treatment of PCV as they show a mild blocking of VEGF and a strong anti-inflammatory effect^24^. Steroids are also known to enhance the barrier function of vascular walls^25^. Intravitreal corticosteroids are proven to be beneficial in managing macular edema secondary to diabetes^26^, uveitis^27^ and retinal vein occlusion^28^, in which inflammation predominates. We postulate that intravitreal dexamethasone, in conjunction with PDT and aflibercept, will be a more effective treatment resulting in faster visual rehabilitation and more rapid reduction of macular edema when treating PCV. This study therefore aims to explore the treatment efficacy of using PDT, aflibercept and dexamethasone in treating PCV. To the best of our knowledge, there are no reports to date on using this specific combination of PDT with aflibercept and dexamethasone in the treatment of PCV.

## Methods

### Patients

This study is a prospective, consecutive case series. All cases were examined and treated by an independent ophthalmologist (D.W.). Seventeen eyes of 13 patients diagnosed with treatment naïve PCV were recruited in a consecutive manner. The study adhered to the tenets of the Declaration of Helsinki. The recruitment period was between 20 Dec 2013 to 22 May 2014 at a single institute (The Hong Kong Ophthalmic Associates, HKOA). The study recruitment and all procedures were approved by the HKOA review panel; informed consent was obtained from all subjects.

### Treatment regimen and data collection

Patients were treated with a combination of PDT, intravitreal aflibercept 2 mg and intravitreal dexamethasone 600 μg/0.15 ml. The first dose of aflibercept and the single dose of intravitreal dexamethasone were given separately on the same day following PDT application; anterior chamber paracentesis was performed to prevent intraocular pressure spikes. Further doses of aflibercept were given on an as-needed basis.

Full fluence PDT was adopted. All patients received a 6 mg/m^2^ infusion of verteporfin for 10 minutes, followed by diode laser (689 nm) 5 minutes later applied to the area of leaking choroidal polyps and abnormal vasculature with an intensity of 600 mW/cm^2^ for 83 seconds. The entire lesion was covered by the laser spot with additional 500 *μ*m covering the borders on each side. One dose of aflibercept was given followed by further doses at monthly intervals if there were signs of persistent or recurrent disease.

The primary outcome of the study was the BCVA at 6 months and 1 year, while secondary outcome included central foveal thickness (CFT), regression of polypoidal lesions, as well as presence or partial regression of the branching vascular network (BVN). The GLD of branching vascular network and polyp size were measured on ICG images using electronic caliber. Polyp size was measured with the greatest linear diameter. Measurement of polyp size and branching vascular network was performed by an investigator who was blinded of all treatment outcome results.

### Investigations and follow up examinations

Patients were seen at 1 week, 1month, 3 months, 6 months and 1 year after the triple therapy treatment. At baseline, all patients underwent best-corrected visual acuity testing, detailed dilated fundus examination, spectral domain-optical coherence tomography (SD-OCT, HRA-2, Heidelberg Engineering, Germany), fundus photography, fluorescein angiography (FA) and ICG (HRA-2, Heidelberg Engineering, Germany). Best corrected visual acuity assessment was performed at every visit. The OCT examination (for documentation of central macular thickness) was performed at baseline, 1 week, 1 month, 3 months, 6 months and at 12 months post initiation of treatment. Fundus angiography and ICG were performed at baseline, 3 months, 6 months and 12 months.

BCVA was measured in a well-illuminated room with optimal refractive correction and standard 6 meter Snellen visual acuity chart. BCVA was converted to the log minimum angle of resolution (logMAR) for analysis. Diagnosis was based on clinical examination showing occasionally the presence of orange-red polyp lesions as well as ICG showing the presence of branching vascular network and/or polypoidal lesions.

### Statistical analysis

Clinical outcomes including BCVA (logMAR) and central foveal thickness were recorded as continuous variables. Differences between these variables at baseline and post treatment outcome were compared using the paired Student *t*-test; a p-value of less than 0.05 was considered statistically significant. Univariate linear regression was used to determine predictive factors for anatomical and visual outcomes, respectively, with statistical tests of p-value <0.05 being considered significant. Statistical analysis was performed with IBM SPSS statistics 18.0 software for Windows (SPSS/IBM Corporation, Chicago, IL, USA).

## Results

Seventeen eyes of 13 patients were recruited (4 females and 9 males). The mean age was 69 years with a range from 43 to 84 years. The baseline clinical information is shown in Table 1. At baseline, the mean number of polyps at presentation was 2.58 +/− 1.2, mean size of polyp was 286 μm +/− 109 μm, 12 eyes (70.5%) had a pigment epithelial detachment (PED), and the mean GLD of the branching vascular network was 1767 μm +/− 1620.

### One-week result

**Figure 1:**
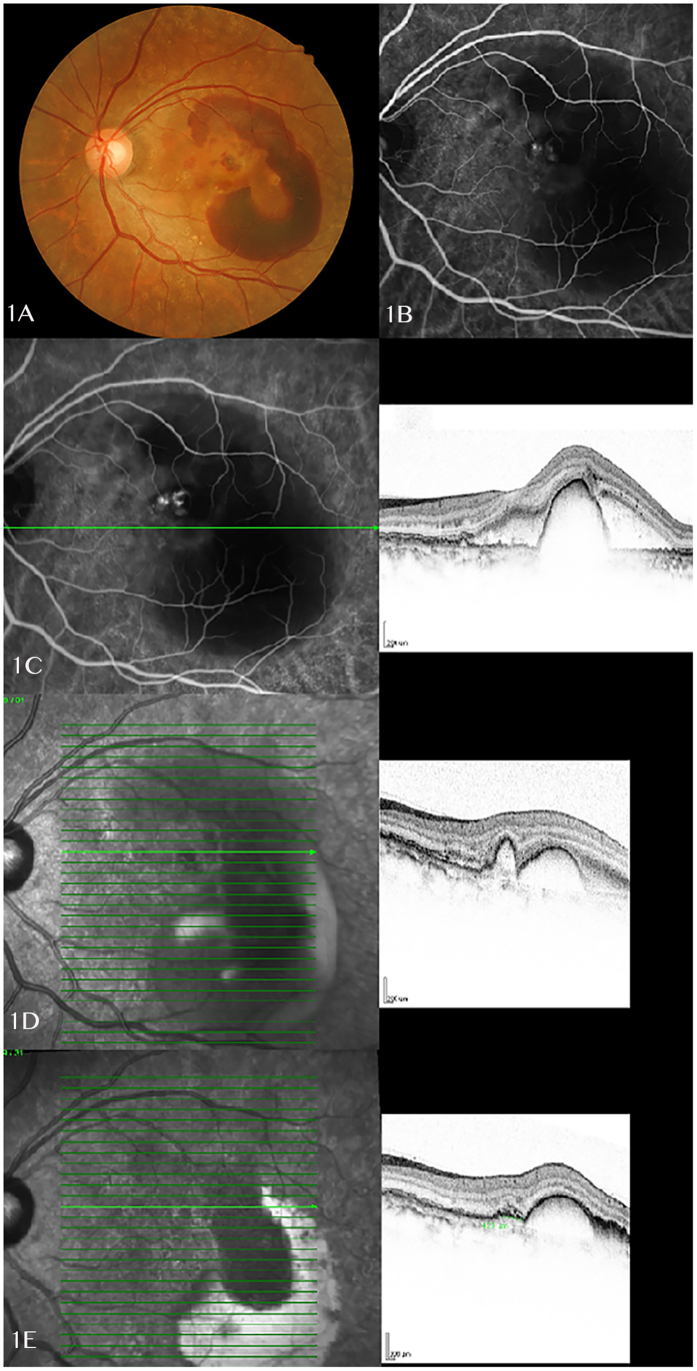
(**A**) A 57 years old female presented with left eye massive submacular haemorrhage and large pigment epithelial detachments. (**B**) ICG examination on the same day showed a cluster of polyps at the edge of notched PED, with a kidney-shaped cyanine dye blocked by the submacular haemorrhage. (**C**) OCT B-scan image showed a central dome-shaped PED with presence of subretinal fluid. Only one OCT image was documented at baseline which shows different sections compared to follow up scans. (**D**) Repeated OCT examination showed largely subsided subretinal fluid and reduced size of PED at 1 week after the triple therapy treatment. B-scan image showed a reduced size of dome-shaped PED and a continuous small PED corresponding to the polypoidal lesions on ICG. (**E**) Infrared imaging and OCT B-scan showed further improvement of disease at one month post treatment, especially reduction in the anterior protrusions of a highly reflective RPE line. (ICG: indocyanine green; OCT: optical coherence tomography; PED: pigment epithelial detachment; RPE: retinal pigment epithelium).

At one week after the first treatment by triple therapy, the mean visual acuity improved to 0.523 logMAR +/− 0.37 (Snellen 20/70), which is significantly improved when compared to baseline 0.651 logMAR +/− 0.38 (Snellen 20/80-20/100; p = 0.04). The anatomical structures also showed significant improvement with CFT reduced to 277 μm +/− 38 from baseline of 394 μm +/− 71 (p = 0.043). Figure 1 shows a case illustration of the quick visual and anatomic response after treatment.

### One-month result

At one month after the therapy, mean visual acuity further improved to 0.363logMAR +/− 0.382 (Snellen 20/50) while CFT reduced to 292 μm +/− 77. Both values showed significant improvement from baseline with p-value of <0.001 and 0.004, respectively. The visual and anatomic responses were significantly improved as early as 1 week post treatment and continued to improve 1 month post treatment.

### Six-month results

Six months after the initiation of triple therapy, the visual acuity remained stable at 0.377logMAR+/−0.440 (Snellen 20/50; p-value 0.005 compared to baseline). The CFT remained stable at 259 μm +/− 54 (p-value 0.012 compared to baseline). ICG showed resolution of the polypoidal lesion in 13 eyes (82%). The branching vascular network showed partial resolution in 11 eyes (64.7%), with the mean greatest linear diameter of 1116 μm +/− 940, and was reduced significantly when compared to baseline (p = 0.016). In this study none of the patients showed complete resolution of the branching vascular network.

### 1-year results

At 1 year after the initiation of triple therapy, the majority of cases remained stable. ICG results of all patients were stable and remained unchanged compared to 6-months ICG findings except two patients (two eyes), who suffered from recurrence of disease. The two patients (two eyes; 12%) with recurrence, reported new onset symptoms at 6 months and 8 months, respectively. The two cases were treated with an additional session of combined therapy consisting of further PDT, intravitreal aflibercept and intravitreal dexamethasone. The average visual acuity at 1 year was 0.35 logMAR +/− 0.41(Snellen 20/40), which was stable and showed no significant change from 6 months onwards (p-value 0.287). The CFT at 1 year was 280 μm +/− 59 and was similar to 271 μm+/− 49 recorded at 6 months (p = 0.47). Twelve eyes (70.5%) had complete resolution of PED.

### Prognostic clinical factors

Univariate linear regression was performed for baseline clinical factors which could affect the visual acuity outcome. Baseline factors included age, baseline visual acuity, baseline CFT, number of polyps, size of polyps, presence of PED, GLD size, PED regression at 6 months and polyp regression at 6 months. The significant outcomes are shown in Table 2. Among all baseline clinical factors, the age, the baseline visual acuity and the presence of PED were found to affect the final visual outcome. A younger age, a better baseline visual acuity and the presence of PED were good prognostic factors for the final visual outcome (Table 2). None of the patients developed cataract or increase in IOP that required glaucoma medication treatment.

## Discussion

The aim of this study was to investigate the efficacy of combining PDT, aflibercept and dexamethasone as an alternative treatment for PCV. The rationale for using this treatment is to achieve polyp regression, reduction of fluid leakage and inflammation which are all known to occur in PCV pathogenesis. Level 1 evidence from the EVEREST trial has shown that PDT either alone or in combination with ranibizumab is needed for polyp regression^5^. Based on this result, the guidelines for treating PCV include either a combination of PDT with ranibizumab or PDT alone as initial treatment^6^. Our study using triple therapy showed similar polyp regression rates (82%) as the EVEREST study (77.8% with PDT monotherapy and 71.4% with PDT and ranibizumab combination therapy) at 6 months. However, it is noted that the number of patients in both of these studies is small (17 eyes in this study and 20 eyes in each treatment arm of EVEREST) and this study did not have a control group with PDT monotherapy. These rates of polyp regression are also similar to those with aflibercept monotherapy where polyp regression can be as high as 77.7%^20^,^21^,^22^,^23^,^29^,^30^. Polyp regression has also been reported with ranibizumab monotherapy; however, branching vascular network persisted despite treatment^31^. Yamamoto *et al.*, similarly reported good outcome by aflibercept treatment with 55.4% complete polyp regession rate on 90 eyes with PCV^30^. The LAPTOP study, a randomized controlled study comparing treatment outcome of PDT and ranibizumab injections, revealed superior visual acuity outcome by ranibizumab injections (4.5 injections) than PDT applications (1.8 PDT applications) at 1 year^32^. The visual acuity gain in ranibizumab arm is significantly better with average gain of 0.9 lines compared to loss of 0.5 lines in PDT treatment group, highlighting the visual acuity benefits by anti-VEGF monotherapy in PCV cases.

In this study we also noticed a high rate of BVN regression. BVN was reduced in size in 64.7% of cases at 6 months after triple therapy. The mean size of BVN decreased from 1,767 μm to 1,116 μm. The significance in reducing the size of the BVN is not entirely clear. The shrinkage of BVN is most likely attributed to PDT, since a few reports have indicated that aflibercept monotherapy has no effect on the size of BVN^33^,^34^,^35^, and it is unlikely that steroids would have an effect on the BVN. Whether shrinking BVN has an effect on visual acuity outcomes or recurrence rates needs further investigation.

In this study, aflibercept was used as part of triple therapy on the basis of its reported superior efficacy for treating PCV over other anti-VEGF agents^36^. Aflibercept monotherapy was reported to be effective in both AMD cases and PCV cases, with a potentially better visual outcome in PCV cases^37^. Polyp regression is achievable by aflibercept monotherapy with reported polyp regression rates between 69.2% to 77%^30^,^37^. Indeed, aflibercept appears useful both as treatment of refractory cases^13^,^14^,^15^,^16^,^17^,^18^,^19^ as well as primary therapy^20^,^21^,^22^,^23^,^29^. This superior efficacy has been attributed to a broader blocking of many angiogenic cytokines (VEGF-A, VEGF-B and PlGF)^30^,^38^, higher binding affinity^9^,^11^ and superior pharmacokinetics^16^,^39^. The main disadvantage of using aflibercept is the frequent injections required, as well as the subsequent patient burden and cost implications. In this study, the number of aflibercept injections were fewer than those reported in the literature^30^. Yamamoto reported high polyp regression rate using aflibercept monotherapy, which required a mean of 7.1 injections in the first 12-month period^30^. The majority of cases in our study required one dose of aflibercept injection, which is substantially less than the 4–7 injections needed with aflibercept monotherapy^20^,^21^,^22^,^23^,^29^,^30^.

Dexamethasone is not routinely used in the treatment of PCV. However, we believe that early restoration of normal macular thickness can be aided by dexamethasone. Intravitreal dexamethasone has been proven useful in the treatment of macular edema secondary to a variety of retinal diseases^40^,^41^. Dexamethasone has a mild effect in blocking VEGF production and a strong anti-inflammatory effect^24^. It also inhibits leukostasis^42^ and can enhance the barrier function of vascular endothelial cell tight junctions^25^. Triple therapy by using PDT, anti-VEGF agents and subtenon steroid therapy has been reported previously. Sakai *et al.* showed that using subtenon steroid injection in addition to combined PDT and anti-VEGF reduced the total number of anti-VEGF injections from a mean of 5.26 to 3.95 in the first year of treatment^43^. Beneficial results by triple therapy when compared to PDT alone were also shown by Nakata *et al.* in a study of 40 retrospective cases. Combined treatment with PDT, bevacizumab and triamcinolone was shown to have longer re-treatment free period, fewer recurrences and potentially fewer subretinal haemorrhages^44^. Severe retinal pigment alternation can occur 1–3 months after uncomplicated PDT application^45^. However, a study period of 1 year may be too short to evaluate the effect of dexamethasone with respect to the long-term protection of RPE atrophy.

Previous randomized control trials on neovascular AMD (rather than PCV) have shown no superior effect of combining PDT with ranibizumab and dexamethasone on visual acuity outcomes. The RADICAL study, however reported significantly fewer re-treatments required in the triple therapy group. Our study on patients with PCV did however show a good response in both visual function and macular thickness as early as one week post triple therapy. The visual acuity improved from 0.651logMAR to 0.523logMAR while central foveal thickness reduced from 394 μm to 277 μm.

In treating PCV, polyp regression, inflammation reduction and VEGF down-regulation are all important. Successful treatment should result in early reduction of macular thickness and subsequent restoration of visual function. This study demonstrates that triple therapy achieves polyp regression with comparable rates to previous studies. We also found rapid reduction of macular thickness and thereby early rehabilitation of vision. Different recommendations exist in the current literature for the treatment of PCV. PDT is recommended in the EVEREST study, due to the high polyp regression rate^5^. On the other hand, the LAPTOP study emphasizes good visual outcome by ranibizumab treatment^32^. An individualized regime, taking into consideration the prognostic factors of PCV including the location of polyps, severity of leakage and the site of abnormal vasculature, could also be considered^46^. To the best of our knowledge, this is the first report demonstrating the safety and efficacy of treating PCV with combination of photodynamic therapy, aflibercept and dexmanethasone. However, the major limitations of this study include a small sample size and the lack of control arms. More data would be preferable in demonstrating the effectiveness of triple therapy. Further studies are needed to compare monotherapy of different anti-VEGF agents with or without additional PDT, as well as to evaluate the value of adjuvant intravitreal steroids in larger randomized controlled clinical trials.

## Additional Information

**How to cite this article:** Ho, M. *et al.* Treatment of polypoidal choroidal vasculopathy by photodynamic therapy, aflibercept and dexamethasone triple therapy. *Sci. Rep.* **6**, 36870; doi: 10.1038/srep36870 (2016).

**Publisher’s note:** Springer Nature remains neutral with regard to jurisdictional claims in published maps and institutional affiliations.

## Figures and Tables

**Table 1:** Demographic data and baseline clinical characteristics of patients harbor with polypoidal choroidal vasculopathy.

	Mean +/− SD (range)
No of patients	13
No of eyes	17
Age; years	69 +/− 16.13 (43–84)
Gender; Male	9 (69%)
Baseline visual acuity; logMAR	0.65+/− 0.38 (0–1.3)
Baseline foveal thickness	394.7 μm +/− 70.6 (229–553)
No. of polyps	2.58 +/− 1.2 (1–4)
Greatest linear dimension of branching vascular network	1767 μm +/− 1620 (378 μm–4550 μm)
Size of largest polypoidal lesion	286 μm+/−109 (130–370)
Presence of pigment epithelial detachment	12 eyes (70.5%)

SD: standard deviation, logMAR: log minimum angle of resolution.

**Table 2:** Univariate linear regression analysis for visual outcome.

Predicting variables	Standardized Regression coefficient	Adjusted R square	p-value
Age	0.811	0.633	<0.001*
Baseline visual acuity (logMAR)	0.698	0.450	0.003*
Presence of PED	−0.943	0.880	<0.001*

*Statistically significant, logMAR: log minimum angle of resolution, PED: pigment epithelial detachment.
